# Chronic myelogenous leukemia on target

**DOI:** 10.1002/cam4.1604

**Published:** 2018-06-14

**Authors:** Veronika Némethová, Filip Rázga

**Affiliations:** ^1^ Department for Biomaterials Research Polymer Institute of the Slovak Academy of Sciences Bratislava Slovakia

**Keywords:** antisense therapy, BCR‐ABL1, chronic myelogenous leukemia, selective interaction, target recognition

## Abstract

Chronic myelogenous leukemia (CML) is commonly treated with tyrosine kinase inhibitors (TKIs) that inhibit the pro‐leukemic activity of the BCR‐ABL1 oncoprotein. Despite the therapeutic progress mediated by TKI use, off‐target effects, treatment‐induced drug resistance, and the limited effect of these drugs on CML stem cells (SCs) are major drawbacks frequently resulting in insufficient or unsustainable treatment. Therefore, intense research efforts have focused on development of improved TKIs and alternative treatment strategies to eradicate CML SCs. Alongside efforts to design superior protein inhibitors, the need to overcome the poor therapeutic effect of TKIs on CML SCs has led to a renaissance of antisense strategies, as they are reported as effective in more primitive cell types. Despite the greater drug design flexibility offered by antisense sequence variability and remarkable chemical improvements, antisense drugs exhibit unacceptable levels of off‐target effects, precluding them from large‐scale clinical testing. Recent advances in antisense drug design have led to a pioneering mRNA recognition concept that may offer a helping hand in eliminating off‐target effects, and has potential to bridge the gap between research and clinical practice.

## 1 COMMENTARY

The management of chronic myelogenous leukemia (CML) has undergone profound evolution over a relatively short period of time. Since its identification, the BCR‐ABL1 oncoprotein, a hallmark of CML cells, has become the primary molecular target for the development of selective therapeutics for patients with CML.^1^ On 28 May 2001, TIME magazine proudly proclaimed “there is new ammunition in the war against cancer—revolutionary new pills like Gleevec combat cancer by targeting only the disease cells.”^2^ Gleevec, the first tyrosine kinase inhibitor (TKI) selectively targeting BCR‐ABL1, remarkably altered treatment paradigms and, compared to former nonselective chemotherapy regimens, contributed dramatically to substantially prolonged survival times and improved quality of life for patients with CML.^3^, ^4^, ^5^, ^6^, ^7^ However, 17 years of clinical experience with TKIs has demonstrated that, despite their apparent therapeutic benefit, these drugs are not entirely selective for CML cells, neither are they sufficiently specific for BCR‐ABL1. Lack of selectivity, various off‐target effects, hematological and nonhematological toxicity, and treatment‐induced drug resistance are recurrent and persistent shortcomings of treatment with TKIs that often result in inferior patient outcomes, require switching to an alternative TKI, may force discontinuation of treatment, or cause adverse events, contributing to decreased quality of life.^8^, ^9^, ^10^, ^11^, ^12^, ^13^, ^14^ Moreover, TKIs have limited therapeutic effect towards CML stem cells (SCs)^15^, ^16^, ^17^, ^18^, ^19^, ^20^, ^21^, ^22^, ^23^, ^24^, ^25^, ^26^; therefore, the majority of patients continue to take these drugs indefinitely, and rarely achieve complete recovery. Nevertheless, there are suggestions that a permanent cure can be induced by TKIs, based on results of TKI stop studies,^27^ which reported that, after deliberate TKI cessation in a strictly clinically defined cohort of patients with CML, half remained free from disease relapse after 2 years of follow‐up. Importantly, because of the restricted detection limit of current methods for monitoring minimal residual disease, it is extremely difficult to discriminate clearly between latent CML and complete cure from the disease. The pitfalls described above highlight the scope for improvement and suggest room for curative treatment approaches with a primary focus on CML SCs and on causal genetic aberrations that trigger leukemogenesis.

A completely different treatment approach is offered by antisense strategies that enable control of disease‐associated genes through interaction with mRNA.^28^, ^29^, ^30^, ^31^, ^32^ In theory, any disease associated with a well‐defined genetic aberration is amenable to mRNA intervention, and antisense systems allowing specific suppression of *BCR‐ABL1* mRNA rapidly became a potential treatment alternative for patients with CML. Compared to the limited, structure‐based design of protein inhibitors, antisense strategies take advantage of far greater target sequence variability for the design of specific antisense drugs. The benefits of sequence‐mediated specificity, allowing selective therapeutic intervention in CML cells, are so great that antisense strategies may be considered one of the most promising future pharmacological prospects for CML treatment since the approval of TKIs.

Interestingly, despite the evident therapeutic potential of antisense strategies, only a minimal number of antileukemic drugs exploiting mRNA interference have reached clinical trials.^29^, ^33^ Biodegradation, limited ability to penetrate cell membranes, and numerous off‐target effects are major obstacles hindering the introduction of antisense therapeutics into clinical practice. The difficulties stemming from biodegradation and insufficient cell membrane penetration have been successfully approached using a variety of nucleotide chemical derivatives.^34^, ^35^, ^36^ By contrast, the issue of unwanted interactions with off‐target mRNAs remains a key unresolved obstacle to smooth clinical translation of this therapeutic concept.^37^

Antisense systems for *BCR‐ABL1* suppression developed to date, 2 of which have even been tested in clinical trials,^38^, ^39^ are based on single antisense oligonucleotides binding to their complementary mRNA sequence spanning the *BCR‐ABL1* gene fusion junction.^40^, ^41^, ^42^, ^43^ Despite successful silencing of *BCR‐ABL1*, in the vast majority of studies, binding to off‐target mRNAs resulted in nonselective effects also in *BCR‐ABL1*‐negative cells. Interference with nontargeted homologous mRNAs, and consequent suppression of proteins outside of therapeutic intent, is a source of clinically relevant side effects and adverse events. Hence, addressing the issue of insufficient selectivity could significantly accelerate the entry of antisense strategies into clinical practice, and innovative solutions for elimination of this phenomenon are in high demand. A promising target recognition concept was recently reported with the intention of addressing this fundamental problem,^44^ and, if proven successful, it could substantially reduce, or even eliminate, undesired off‐target effects.^37^ In the context of CML, the concept may facilitate specific *BCR‐ABL1* mRNA recognition, and hence selective therapeutic intervention, exclusively in CML cells.

**Figure 1:**
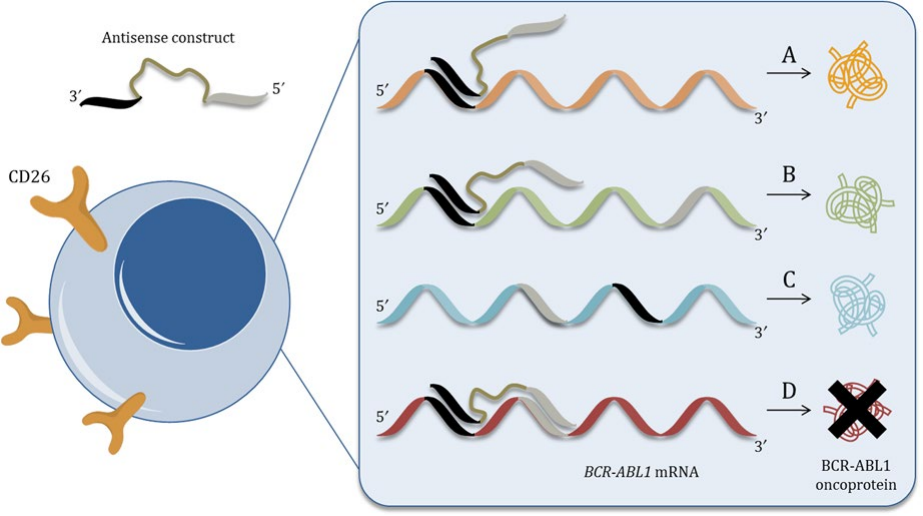
Illustration of the antisense construct and its binding to various mRNAs. A, Partial binding of the antisense construct to an inadvertent mRNA due to the absence of one of the target sequences; B, Partial binding of the antisense construct to an inadvertent mRNA due to the inappropriate distance of target sequences; C, No recognition of the target sequences due to their inappropriate orientation; D, Full recognition of both target sequences and stable binding of the antisense construct to the target *BCR‐ABL* mRNA. (A‐C) Represent unstable binding modes of the antisense construct and are expected to have a negligible effect on mRNA suppression. (D) Represents the thermodynamically and energetically preferred binding mode of the antisense construct leading to selective suppression of target mRNA. CD26 cell surface marker^47^ allows for direct targeting of CML SCs

The solution addresses the basic design of the antisense construct, which is engineered to recognize the target mRNA via 2 antisense oligonucleotides connected by a size‐specific linker (Figure 1).^44^ Simultaneous sequence‐specific binding of individual antisense oligonucleotides to complementary sequences of partner *BCR* and *ABL1* mRNAs flanking the gene fusion junction can occur only when these sequences are separated from one another by a specific distance, which is defined by the linker. This simple but strict requirement significantly reduces the probability of stable interference with off‐target mRNAs, and thereby may provide dramatically enhanced selectivity towards CML cells.

This selective antisense strategy is based on the assumption that partial interaction of the antisense construct with wild‐type *BCR* and *ABL1* gene sequences, or with homologous sequences of off‐target mRNAs, will be thermodynamically unstable and energetically unfavorable because of the increased degree of conformational freedom and the absence of cooperative hydrogen bonds, respectively (Figure 1A‐C).^44^ Furthermore, any stable intermolecular interactions between the antisense construct and partially complementary mRNAs are even more improbable. Such nonspecific interactions are therefore expected to have negligible effects on the suppression of nontarget mRNAs. Consequently, selective interference with *BCR‐ABL1* mRNA (Figure 1D) will ensure therapeutic intervention only in CML cells, substantially reducing the potential for side effects and adverse events. Moreover, as antisense systems are reported to function, not only in actively proliferating cells, but also in more primitive CML cells,^45^, ^46^ there is also a real prospect of suppression of *BCR‐ABL1* mRNA in CML SCs. The hope for a permanent cure for CML has been further advanced by recent reports of the phenotype of CML SCs.^47^, ^48^, ^49^ The synergy of selective therapeutic intervention and active targeting could facilitate disease eradication with minimal off‐target effects. It is important to note, however, that eradication of CML SCs may not be trivial or straightforward, since studies on these cells indicate the presence of complex *BCR‐ABL1*‐independent signaling pathways which can contribute to escape from drug‐induced apoptosis.^15^, ^50^ Several parallel targets, such as *FOXO*,* BCL6*, and *CXCR4*, or proteins involved in the WNT, Hedgehog and JAK signaling pathways have already been identified as relevant in this respect.^51^ In line with the multiple targeting approaches emerging in modern drug R&D, simultaneous selective blocking of the most relevant players may thus represent the future strategy in more effective fight against CML SCs.

The vision of CML SCs eradication got even more realistic contours after the cell surface marker CD26 has been identified^47^ as discriminatory from healthy hematopoietic SCs. However, the CD26 surface marker is not specific only to CML SCs, but is expressed also by other, nonhematopoietic cell types. This has obviously led to an objective lag in testing anti‐CD26 therapies. Noteworthy though, in case of a drug with exquisite selectivity toward the intracellular target, its overconcentration in the vicinity of a specific cell population via active targeting allows to focus the therapeutic intervention in CD26+ SCs, without undesired effect in CD26+ cells absenting the intracellular target. Comprehensive follow‐up research is understandably needed to support this presumption.

Considering the history and evolution of treatment strategies for CML, and irrespective of the tortuous path toward treating CML without side effects, the therapeutic potential of the novel mRNA targeting approach described above is clearly worthy of thorough investigation. The progressive nature of this antisense concept is underlined by its theoretical applicability to any disease where the presence of a particular protein has causal role in pathophysiology.^44^ This concept may thus serve as a universal platform for the future design of antisense therapeutics.

The extent to which this novel strategy will be able to alter the *status quo* in oligonucleotide‐based therapeutics will emerge over time. The fascinating possibility of leukemia treatment using a specific agent that can completely spare healthy cells remains a potent stimulus to the continued exploration of this approach and would facilitate its implementation into clinical practice.

## CONFLICT OF INTEREST

The authors declare no potential conflicts of interest.
